# Growth and Nutritional Status of Phenylketonuric Children and Adolescents

**DOI:** 10.1186/s12887-022-03715-2

**Published:** 2022-11-17

**Authors:** Mina Ahmadzadeh, Golbon Sohrab, Mohammadreza Alaei, Hassan Eini-Zinab, Behzad mohammadpour-Ahranjani, Samira Rastgoo, Zahra Namkhah

**Affiliations:** 1 Department of Clinical Nutrition and Dietetics, Faculty of Nutrition Sciences and Food Technology, National Nutrition and Food Technology Research Institute, Shahid Beheshti University of Medical Sciences, Tehran, Iran; 2 Department of Pediatric Endocrinology, Faculty of Medicine, Shahid Beheshti University of Medical Sciences, Tehran, Iran; 3 Department of Pediatric Cardiology, Bahrami Hospital, Tehran University of Medical Sciences, Tehran, IR Iran; 4 Department of Clinical Nutrition, School of Nutritional Sciences and Dietetics, Tehran University of Medical Science, Tehran, Iran; 5 Department of Community Nutrition, Faculty of Nutrition Sciences and Food Technology, National Nutrition and Food Technology Research institute, Shahid Beheshti University of Medical Sciences, Shahid Beheshti University of Medical Sciences, Tehran, Iran

**Keywords:** Phenylketonuria, Phenylalanine, Nutritional status

## Abstract

**Background:**

The goal of this study was to assess the anthropometric and biochemical parameters of children and adolescents with phenylketonuria (PKU).

**Methods:**

The participants in this cross-sectional study ranged in age from four to 18 years old. Biochemical markers such as vitamin B12, folic acid, iron, ferritin, calcium, 25-hydroxy vitamin D3, zinc, plasma phenylalanine (Phe) and tyrosine (Tyr) levels in blood were evaluated, as well as demographics and anthropometric measurements. A three-day dietary recall questionnaire was completed by all individuals.

**Results:**

80% (64) of the 80 patients (42 females, 52.5%) had typical PKU. Consanguineous marriages were found in 57.5% (46) of the patients’ parents. According to the height for age index, 17.5% of the study group (n = 14) were short or very short. According to age-related weight and body mass index (BMI), 37.5% (n = 30) and 43.8% (n = 35) of people are obese or overweight, respectively. Biochemical tests revealed increased vitamin B12 levels and 25-hydroxy vitamin D3 deficiency in 35% (n = 28) of the patients, insufficient folic acid in 12.5% (n = 10), and elevated phenylalanine levels in 70.3% (n = 45) of children under 12 years old, and adolescents 62.5% (n = 10). A high Phe intake (OR = 4.44, CI %95 = 1.27–15.57) is a risk factor for obesity and overweight.

**Conclusion:**

Patients with PKU had a high rate of overweight and obesity. PKU patients who are overweight or obese do not differ from normal-weight patients in terms of dietary intake or laboratory findings (except for serum iron levels). One-third of patients with phenylketonuria were vitamin D deficient and had a BMI/A index of overweight/obese. It is recommended to use special medical food to help solve energy and nutrient deficiencies.

## Introduction

Phenylalanine hydroxylase (PAH), is a cytosolic homotetramer [1] and monooxygenase enzyme [2] that hydroxylated phenylalanine [3] using tetrahydrobiopterin (BH4) as a cofactor [4]. Deficiencies in this hepatic enzyme cause a rare genetic disorder commonly known as phenylketonuria (PKU) [5]. Because phenylalanine hydroxylase catalyzes the conversion of phenylalanine (Phe) into tyrosine (Tyr), PKU causes high Phe levels in the blood and tissues [6]. Untreated high Phe levels can harm the brain and nervous system by disrupting synapses and affecting neurotransmitter metabolism [2]. As a result, more persons in society have physical, mental, and low intelligence quotient (IQ) problems, which places a heavy financial strain on families and the healthcare system [7, 8]. Severe mental retardation, epilepsy, growth retardation, psychological issues, hyperactivity, progressive microcephaly, overall weakness, dermatitis, urine odor of a dead mouse, hypopigmentation, blond hair, and iris blue are all symptoms of this condition [1, 9–16]. The frequency of PKU varies greatly between ethnic groups and geographical areas [17]. In Europe, the average live birth prevalence is 1: 10,000 [18]; in the United States, the average is 1 in 15,000; and in Iran, one in 8000. Consanguinity among carrier couples is the leading risk factor for PKU due to its autosomal recessive inheritance [19]. In areas with a high prevalence of consanguineous marriages, this disease is more prevalent [12, 20, 21].

In most cases, treatment begins during the first two weeks of life [2]. The goal of phenylketonuria treatment is to reduce the level of Phe in the blood by reducing the Phe intake. The level of phenylalanine in the blood is the most important factor in determining treatment [4]. PKU is treated with a low-phenylalanine diet for the rest of the patient’s life, either alone or in combination with medicine [22]. The diet’s guiding principles often include avoiding meat, dairy products, eggs, beans, and nuts while consuming specific amounts of vegetables, fruits, and medical foods [23]. Objective blood phenylalanine ranges are: 120 to 360 µmol/L (2–6 mg/dL) for patients up to 12 years old and 120 to 600 µmol/L (2–10 mg/dL) for patients ≥ 12 years of age [22]. An increased intake of carbs and sweets, which has been linked to weight gain in these individuals, has been identified as a serious drawback of this type of diet in research [2, 14]. On the other hand, special dietary intakes and severe restrictions on protein intake can result in nutritional deficiency [24]. In these patients, low levels of antioxidant micronutrients have been linked to poor mental function and psychosocial issues [25]. Consequently, anthropometric indicators, clinical indicators of nutrient deficiency, nutrient intake, and biomarkers to identify excess or lack of nutrients are needed for nutritional follow-up [26]. In recent years, limited studies have examined the nutritional status of these patients [12, 27, 28]. Some studies have examined the status of macro-and micronutrients [29–32], and few have analyzed anthropometric indices and body composition [33–36].

In Iran, the government provides a formula for metabolic patients. To assess the nutritional state of these patients and improve nutritional management, it is critical to accurately measure nutrition and environmental markers. The purpose of this study was to determine the nutritional status and growth of children and adolescents with PKU in Tehran, Iran.

## Materials

### Participants

The data for this cross-sectional study were collected from April to September 2020, and samples with an age range of 4 to 18 years old were included. PKU patients (n = 94) at the Mofid Children’s Hospital were studied. Insufficient laboratory data and unwillingness to participate in the trial due to COVID-19 (n = 10) or severe comorbidity (n = 4) led to the exclusion of 14 patients from the study. Except for 12 PKU kids, all were diagnosed within two weeks of birth through newborn screening. These 12 patients ranged in age from 1 to 20 months at the time of diagnosis. Exclusion criteria of the study were: cancer, any serious cardiovascular, hematological, renal, hepatic, neurological, gastrointestinal, endocrine, rheumatism, skeletal, severe infections, skin problems, and any other illness that can severely influence nutritional status. The presented study was approved by The Ethics Committee of the National Nutrition & Food Technology Research of Iran with the ethical code of IR.SBMU.NNFTRI.REC.1398.077. Informed consent was obtained from all participants or their legal guardian. All methods were performed in accordance with the relevant guidelines and regulations.

### Patients’ characterization

Depending on the increasing rate of Phe concentrations in blood, the severity and biochemical phenotypes of the disease can be classified: Phe levels of 120–600 µmol /L as benignant hyperphenylalaninemia (HPA), Phe levels of 600–1,200 µmol /L as mild PKU, Phe levels > 1,200 µmol /L as classical PKU [37].

Demographics (age, gender) and anthropometrics data (weight, height and, [body mass index (BMI)]) were recorded. In this study, the sample’s weight was measured using a digital weighing scale with 100 g of accuracy and their heights were measured with an accuracy of 0.1 cm. The BMI was calculated by dividing the weight in kilograms by the square of the patient’s height in meters. To assess children’s development, Z-scores of W/A [weight for age], H/A [height for age], and BMI/A [BMI for age] were calculated for each patient using WHO Anthro (for children under 5 years old) and AnthroPlus® (for children 5 to 19 years old) software version 3.2.2, (World Health Organization, Geneva, Switzerland). Based on the BMI/A z-score, patients were classified into two groups including overweight/obese (SD ≥ + 1) and normal/underweight (SD < + 1), and the studied parameters were compared in these two groups.

### Laboratory data

Biochemical parameters including serum vitamin B12, folic acid, serum iron, ferritin, calcium, 25-hydroxy vitamin D3, zinc, and levels Phe and Tyr in plasma were measured after two to four hours of fasting blood samples. High-performance liquid chromatography (HPLC) was used to measure plasma Phe levels. The reference range for laboratory tests was determined according to the valid laboratory kit used as follows: plasma Phe (< 12 years): 2–6 mg/dl; plasma Phe (≥ 12 years pld): 2–10 mg/dl; vitamin B12: 200–835 pg/mL; folic acid: 6–20 ng/mL; serum iron: 33–140 mg/dL; ferritin: 4-104.2 ng/mL; albumin: 3.8–5.4 µg/dL; 25-hydroxy vitamin D3: 30–100 ng/mL; zinc: 60–129 mg/dL.

### Dietary intake

Face-to-face interviews were used to complete a three-day dietary recall questionnaire for all patients. The participants and their legal guardians were present while collecting all the data. Then using these questionnaires and the Nutritionist IV software (First databank; Hearst Corp., San Bruno, California, USA) data for nutritional factors (such as daily energy intake, carbohydrate, fat, protein, phenylalanine, and tyrosine) were.

### Statistical analysis

SPSS software version 26 was used for statistical analysis of the results. The Shapiro-Wilk test was adopted to check the normality of the parameters. The logarithm on the base of ten was used for the normalization of non-normal distributed variables, including age, dietary intake of protein, calories, carbohydrate and Phe. The characteristics of demographic, social, and anthropometric indicators of the participants were described using descriptive statistics based on the mean and standard deviation (for quantitative data) and percentages and numbers (for qualitative data). Then using Chi-square tests the association between these variables was investigated. For both normal and non-normal distributed variables, the Independent Sample T-test or Mann–Whitney U test was used to compare dietary intake and laboratory results in BMI groups. The effects of daily calorie, protein, fat, carbohydrate, and phenylalanine consumption on obesity status were assessed using logistic regression. At p < 0.05, the results were declared significant.

## Results

Out of 94 children and adolescents with PKU, 80 persons (85.1%) took part in this study. Consanguineous marriages were found in 46 parents (57.5%) of the patients.

Table 1 shows the nutritional status of the participants. According to the H/A index, 17.5% of the study population (n = 14) were short or extremely short. Also, with regards to the W/A and BMI/A indices, 37.5% (n = 30) and 43.8% (n = 35) of people are overweight or obese, respectively.

**Table 1:** Anthropometric indices weight for age, height for age and BMI-for-age in PKU patients between 4 and 18 years

variables	All PKU (N = 80)	z-scores		
		-1 SD - +1 SD	< -1 SD	+1 SD <
Height/age z-scores	0.21 ± 1.40	44 (55%)	14 (17.5%)	22 (27.5%)
Weight/age z-scores	0.64 ± 1.06	46 (57.5%)	4 (5%)	30 (37.5%)
BMI/age z-scores	0.70 ± 1.08	38 (47.5%)	7 (8.8%)	35 (43.8%)

Data are presented as n (%); PKU: Phenylketonuria; SD: standard deviation

Table 2 compares the results of some demographic data, nutritional consumption, and biochemical tests performed on patients using BMI z-score categories. The participants were split into two groups. Seven persons were underweight and 38 persons had normal weight in the first group. Twenty-six participants in the second group were overweight, and nine were obese. The average age of the participants was 9.01 ± 3.64 years (4–18 years), with 42 (52.5%) females and 38 (47.5%) males. There was a significant difference in age between the BMI z-score categories, the mean age of the first group (normal and underweight people) was higher (p < 0.05), but no significant variation in other demographic data between the BMI z-score categories was detected. Regarding nutritional intake, there was no statistically significant difference between the two groups. The overweight group had a significantly decreased mean serum iron level (p < 0.05). Other laboratory parameters were not significantly different between the groups (p > 0.05).

**Table 2:** Comparison of mean ± SD and frequency distribution of Patient’s laboratory findings and dietary intake of PKU patients

variables	All PKU	Underweight /Normal	Overweight and Obese	p-value
	n = 80	n = 45	n = 35	
Age (y)	9.01 ± 3.64	9.81 ± 4.11	7.97 ± 2.65	0.043
Gender (female)	42 (52.5%)	23 (51.1%)	19 (54.3%)	0.778^b^
Time of diagnosis (≤ 2week)	68 (85%)	36 (80%)	32 (91.4)	0.156^b^
Phenotype (classic)	64 (80%)	36 (80%)	28 (80%)	1
Consanguineous marriage	46 (57.5%)	23 (51.1%)	23 (65.7%)	0.13
**Dietary intake**				
Energy (kcal)	1323.29 ± 392.79	1373.85 ± 435.07	1258.28 ± 325.25	0.197
Protein (g)	51.95 ± 16.03	53.22 ± 16.54	50.31 ± 15.43	0.423
Fat (g)	41.02 ± 25.25	42.43 ± 29.30	39.22 ± 19.09	0.734^a^
Carbohydrate (g)	193.85 ± 63.97	200.56 ± 66.97	185.21 ± 59.73	0.260
Phe intake (mg)	655.86 ± 663.08	758,94 ± 785.45	523.33 ± 437.10	0.120
**Laboratory data**				
Plasma Phe level (mg/dl)	11.26 ± 7.16	12.17 ± 8.20	10.09 ± 5.43	0.446^a^
Plasma Tyr level (mg/dl)	1.20 ± 0.69	1.21 ± 0.77	1.18 ± 0.58	0.662^a^
Serum albumin level (g/dl)	4.39 ± 0.27	4,40 ± 0.28	4.37 ± 0.27	0.591^a^
Serum iron level (µg/dl)	85.31 ± 34.39	95.15 ± 36.42	72.65 ± 27.18^b^	0.003
Serum calcium level (mg/dl)	10.18 ± 0.40	10.16 ± 0.42	10.20 ± 0.39	0.667
Serum zinc level (µg/dl)	95.35 ± 28.53	96.33 ± 27.72	94.08 ± 29.91	0.729
Serum folic acid (ng/ml)	18.75 ± 12.59	17.02 ± 9.28	20.97 ± 15.74	0.229^a^
Serum vitamin B12 level (pg/ml)	806.03 ± 426.12	741.82 ± 397.30	888.59 ± 452.94	0.127^a^
Serum ferritin level (ng/ml)	43.82 ± 49.51	39.01 ± 19.71	49.99 ± 71.58	0.617^a^
Serum 25-Hydroxy vitamin D3 level (ng/ml)	37.28 ± 20.19	37.07 ± 22.47	37.55 ± 17.12	0.713

Data are presented as mean ± SD, n (%); PKU: phenylketonuria; SD: standard deviation; Phe: phenylalanine; Tyr: tyrosine

^a^ The value is calculated using Mann-Whitney Test ^b^ The value is calculated using Pearson Chi-Square Test

**Fig. 1:**
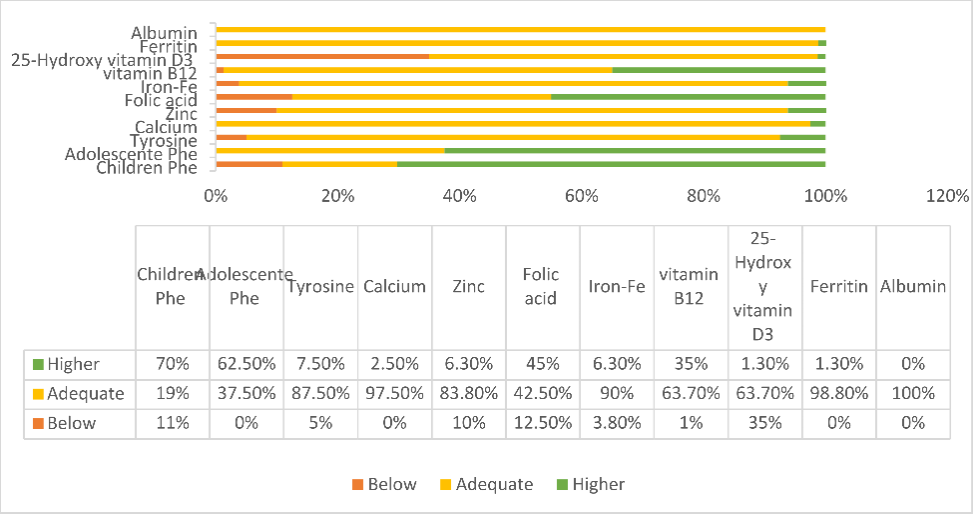
Adequacy of biochemical tests according to reference values of children and adolescents between 4 and 18 years old with phenylketonuria - Pearson’s Chi-square test and Fisher’s exact test: p > 0.05

When comparing the results of the biochemical tests by the reference values, low levels of 25-hydroxy vitamin D3 and folic acid were observed in 35% (n = 28) and 12.5% (n = 10) of the cases respectively. Low levels of vitamin B12, zinc, iron and, tyrosine were observed with lower frequency. Values higher than the reference values most frequently observed for vitamin B12 (35%, n = 28), Phe in children under 12 years old (70.3%, n = 45), and Phe in adolescents (62.5%, n = 10). It was observed that 25% of cases (n = 20) used vitamin D supplementation. Among those who received supplementation, it was observed that they had deficient levels of 25-hydroxy vitamin D3 (14.3%, n = 4). According to Fig. 1, while the BMI z-score category and serum calcium level differed significantly (p < 0.05), the BMI z-score category and other laboratory variables did not (p > 0.05).

Raw and adjusted logistic regression was used to explore the relationship between food intake and the risk of obesity and overweight. Participants were separated into three equal groups based on their food intake. The first group (Q1), which had the lowest consumption of each food, served as a control group against which the other two groups (Q2 = the average intake and Q3 = highest intake) were compared. In this method, three models were extracted. The first model was a crude model. A high intake of calories, carbs, proteins, and fats is not a risk factor for overweight and obesity when age and sex (model 2) and age, sex, and Calories from medical formula (model 3) are taken into account as confounders. However, in model 1, Table 3, high phenylalanine consumption (OR = 4.44 CI percent 95 = 1.27–15.57) is a risk factor for obesity and overweight.

**Table 3:** Odds ratio and 95% confidence interval for the association between dietary intake and overweight/obesity

		Crude	Model 2	Model 3
Protein intake	Q1	1	1	1
	Q2	1.69 (0.57–5.03)	0.90 (0.25–3.25)	0.72 (0.18–2.85)
	Q3	0.85 (0.25–2.55)	0.61 (0.18-2.00)	0.47 (0.13–1.75)
	P-trend	0.19	0.33	0.11
Energy intake	Q1	1	1	1
	Q2	1.70 (0.56–5.08)	1.18 (0.36–3.86)	0.68 (0.10–5.47)
	Q3	1.36 (0.45–4.04)	1.26 (0.40–3.97)	0.97 (0.22–4.29)
	P-trend	0.18	0.33	0.36
Carbohydrate intake	Q1	1	1	1
	Q2	1.70 (0.56–5.08)	1.12 (0.36–3.65)	1.33 (0.39–4.54)
	Q3	1.36 (0.45–4.04)	1.33 (0.43–4.14)	1.38 (0.43–4.34)
	P-trend	0.58	0.94	0.64
Fat intake	Q1	1	1	1
	Q2	1.24 (0.42–3.70)	1.05 (0.34–3.28)	1.71 (0.47–6.22)
	Q3	1.16 (0.39–3.42)	1.13 (0.36–3.54)	1.16 (0.36–3.72)
	P-trend	068	0.23	0.73
Phenylalanine intake	Q1	1	1	1
	Q2	2.20 (0.65–6.72)	2.09 (0.65–6.72)	1.76 (0.47–6.55)
	Q3	2.77 (0.89–8.57)	4.44 (1.27–15.57)	3.74 (0.93–15.02)
	P-trend	0.18	0.02	0.09

Data are presented as OR and 95% CI

Model 2: adjusted for age and sex, Model 3: Further adjustments for caloric intake of medical formula

## Discussion

To the best of our knowledge, this is one of the first studies in the Middle East to examine the relationship between food consumption and nutritional status in children and adolescents with PKU. Our findings suggest that PKU patients who consume more Phe had a higher probability of having a high BMI than those who consume less Phe, but there is no direct association between BMI and calorie, protein, carbohydrate, or fat intake.

Various studies showed growth retardation in children and adolescents with PKU [23, 28], while one of our assumptions in this study was the incidence of obesity in phenylketonuria patients. This hypothesis was tested using the BMI z-score. According to their BMI z-score values, about 44% of patients in this study were overweight or obese, and females had higher BMI z-score values. In a study conducted by Almeida et al. [38] in Brazil, the prevalence of obesity and overweight was 28.5%. Also, in the studies of Camatta et al. [14] and Mazzola et al. [39], the prevalence of obesity and overweight was lower than our study (19.13% and 22%, respectively). In the Ogden et al. study of two to 19 years old in the United States, the prevalence of overweight and obesity was 31.8% [40]. Shakbia et al. also published a cross-sectional study in 2019 entitled Evaluation of anthropometric indices in patients with phenylketonuria. They said that children and adolescents treated with diet from birth had higher weight and body mass index. They reported that this group of children’s overweight was the long-term use of high-carbohydrate and low-protein diets [41]. As a result, the prevalence of overweight and obesity in Brazil and the United States is lower than in Iran. Because of the poor protein consumption of these patients and the high cost of special low protein foods (SLPF) in Iran, carbs, sugars, and fats constitute the majority of the patients’ calories, which may explain the increased prevalence of overweight and obesity in this study. In contrast, in 2009, Eshraghi et al. investigated the nutrition and growth status of Mazandaran children and adolescents with phenylketonuria. Of these, 23.8% of the patients were underweight, and 19% were short. Mazandaran children and adolescents with phenylketonuria risk poor micronutrients needed for proper growth. Researchers have identified the lack of access to food appropriate to the disease as the leading cause of growth retardation in these patients [28].

Almeida et al., who supported the findings of this investigation, found that 88.1% of the sample was sufficient in terms of the H/A z-score [38].

The majority of patients exhibited normal serum levels of albumin, ferritin, iron, calcium, zinc, and tyrosine, according to biochemical tests. Vitamin D deficiency was found in 35% of patients, and high plasma phenylalanine levels indicated poor disease control. Vitamin B12 and folic acid serum levels were high in 45 and 35% of patients, respectively. There was no significant difference between the two BMI groups except for serum calcium levels.

In contrast to our findings, in the Barretto et al. study [42], zinc levels were below the normal values in 37.5% of people. One possible reason is that the zinc bioavailability may be affected by excess fiber, phytates, and other minerals. Also, it seems that due to the restriction of the natural protein diet in these patients and receiving vegetarian diets, zinc absorption has decreased [43]. However, in our study, zinc deficiency was seen in 10% of patients, which is consistent with the results of some previous studies [30, 38, 44].

Iron, vitamin B12, and folate are important for oxygen transport and optimal cognitive function, thus their deficiency can affect brain function [3]. Similar to what was observed in the studies of Crujeiras et al. [29], and Kose et al. [45], vitamin B12 and folic acid deficiency were negligible in our study. 45% and 35% of patients had high serum concentrations of these vitamins, respectively. Similar to the present study, the results of Evans et al. [44] and Almeida et al. [38] studies showed that more than 90% of the persons had adequate and normal ferritin levels.

Compared to the results of the Demirdasa et al. study [30], the apparent lack of vitamin D was observed. While the prevalence of vitamin D deficiency was higher (53.57%) in Kose et al. study [45]. Since vitamin D is involved in neurotransmission, calcium balance maintenance, signaling, and synaptic plasticity, deficiency in this vitamin can increase the effects of inadequate phenylketonuria control [25]. Consequently, its it is imperative to address its deficiency to avoid neurological problems.

There were several major flaws in this study, including that it was cross-sectional and conducted in a single center with a small number of patients, making it difficult to extrapolate its findings to other patients. Because of the small sample size, no causal association between dietary intake and BMI can be inferred as a pilot hypothesis-generating study. Of course, our study had a higher sample size than other earlier studies [23, 28, 30, 39].

Finally, this study looked into the nutritional status of phenylketonuria patients. The implementation of this method has aided in the regulation of blood phenylalanine levels and the prevention of neurological diseases in phenylketonuria patients. According to the findings, nearly one-third of the patients were vitamin D deficient and had a BMI/A index of overweight/obese. Furthermore, the majority of the individuals showed elevated phenylalanine levels in their blood. Evidence suggests that patients with phenylketonuria may experience neurological and psychiatric issues as a result of hyperphenylalaninemia. On the other hand, several micronutrient deficiencies, such as iron, vitamin D, B12, and folic acid, can lead to neurological problems. As a result, we believe that nutritional deficiencies may exacerbate the disease’s severity. PKU patients should follow a phenylalanine-restricted diet with a balanced intake of macronutrients and micronutrients on a regular basis and be monitored for plasma phenylalanine levels. More research is needed on the impact of dietary inadequacies and micronutrient intake on phenylketonuria, as well as evaluating overweight, obesity and other obesity-related concerns in these patients as they age.

## Data Availability

The raw data supporting the conclusions of this article will be made available by the corresponding author, without undue reservation, to any qualified researcher.

## Abbreviations

PKU: phenylketonuria
Phe: phenylalanine
Tyr: Tyrosine
HPA: hyperphenylalaninemia
BMI: body mass index
PAH: phenylalanine hydroxylase
IQ: intelligence quotient
W/A: weight for age
H/A: height for age
BMI/A: BMI for age
